# Distinct Contributions of JNK and p38 to Chromium Cytotoxicity and Inhibition of Murine Embryonic Stem Cell Differentiation

**DOI:** 10.1289/ehp.0800157

**Published:** 2009-04-03

**Authors:** Liang Chen, Jerald L. Ovesen, Alvaro Puga, Ying Xia

**Affiliations:** Department of Environmental Health, University of Cincinnati College of Medicine, Cincinnati, Ohio, USA

**Keywords:** chromium, cytotoxicity, embryonic stem cell differentiation, ERK, JNK, MAPKs, p38, ROS

## Abstract

**Background:**

Potassium dichromate [Cr(VI)] is a widespread environmental toxicant responsible for increased risk of several human diseases. Cr(VI) exposure leads to activation of mitogen-activated protein kinases (MAPKs), including c-Jun N-terminal kinase (JNK)1/2, p38, and extracellular-signal regulated kinase (ERK)1/2.

**Objectives:**

We evaluated the contribution of MAPKs to Cr(VI) toxicity.

**Methods:**

Phosphorylation of MAPKs and their downstream effectors was evaluated by Western immunoblotting; reactive oxygen species were measured by DCFDA (5′,6′-chloromethyl-2′-7′-dichlorofluorescin diacetate) labeling and flow cytometry, and glutathione and glutathione disulfide levels were determined by monochrome graphic spectroflurometer. Cytotoxicity was assessed by the MTS [3-(4,5-dimethylthiazol-2-yl)-5-(3-carboxymethoxyphenyl)-2-(4-sulfophenyl)-2H-tetrazolium] assay and colony formation. Embryoid body (EB) differentiation was evaluated by contracting cardiomyocyte formation, and real-time polymerase chain reaction (RT-PCR) was used for cardiomyocyte-specific and stem-cell-specific gene expression.

**Results:**

Acute treatment of mouse embryonic stem (ES) cells with 50 μM Cr(VI) induced the rapid phosphorylation of JNK, p38, and ERK and their respective downstream transcription factors, c-JUN, activating transcription factor-2, and ELK1. MAPK activation and cytotoxicity induction were partially blocked by pretreatment with the antioxidant *N*-acetyl cysteine. Ablation of the upstream MAP kinase kinase (MAP2K7) in ES cells prevented JNK activation, whereas ablation of MAP2K4 prevented both JNK and p38 activation. Using specific MAPK inhibitors and MAP2K4- and MAP2K7-deficient ES cells, we showed that JNK reduced acute Cr(VI) cytotoxicity, p38 potentiated it, and ERK had no effect. At low submicromolar concentrations, Cr(VI) caused MAP2K4/7-dependent JNK activation and MAP2K4-dependent p38 activation and strongly inhibited contracting cardiomyocyte development in wild-type ES cells, but much less so in *Map2k7*^(−/−)^ cells.

**Conclusion:**

Each MAPK distinctly contributes to chromium toxicity. Whereas JNK prevents and p38 promotes acute cytotoxicity, JNK contributes to optimal inhibition of ES cell differentiation by chromium.

Potassium dichromate [Cr(VI)] is the most toxic form of chromium produced by industrial processes and is a common environmental contaminant (Langard 1990). Humans exposed to Cr(VI) under environmental or occupational settings show increased risk of asthma, nasal septum and skin ulcerations, allergic and contact dermatitis, and respiratory cancers (Salnikow and Zhitkovich 2008). Experimental mice exposed to chromate exhibit acute tissue damage, including testicular lesions, kidney tubular necrosis, and liver toxicity, whereas Cr(VI) ingestion via drinking water leads to increased DNA damage and ultraviolet-induced skin tumor formation in mice (Davidson et al. 2004; Ueno et al. 2001). In addition, Cr(VI) is a developmental toxicant that causes developmental defects, including postimplantation losses, resorptions, reduced fetal weight, and malformations (Junaid et al. 1996; Kanojia et al. 1998).

Particulate Cr(VI) undergoes dissolution on the cell surface, and the chromate oxyanion enters the cells through the nonspecific phosphate/sulfate ion transporters (O’Brien et al. 2003). Once inside the cell, chromate can be rapidly reduced by antioxidants, such as ascorbate, glutathione, and NAD(P)H, to generate reactive Cr(V) and Cr(IV) intermediates that are strong oxidants. The Cr intermediates are subsequently reduced to form the thermodynamically stable Cr(III), which is primarily responsible for DNA adducts formation and mutagenicity (Salnikow and Zhitkovich 2008). On the other hand, Cr(VI) has been shown to activate cellular signaling pathways that in turn lead to either promoting (Salnikow and Zhitkovich 2008; Wakeman et al. 2005; Wang and Shi 2001) or preventing Cr(VI) toxicity (Chen et al. 2002; He et al. 2007).

One group of signaling factors activated by Cr(VI) is the mitogen-activated protein kinases (MAPKs) (Chuang and Yang 2001; Tessier and Pascal 2006). MAPKs are cytosolic Ser/Thr protein kinases, comprising the extracellular signal-regulated kinase (ERK) (ERK1/2), c-Jun N-terminal kinase (JNK) (1/2/3), and p38 (α/β/γ/δ) subfamilies. Activation of the MAPKs is mediated through the specific upstream MAP kinase kinases (MAP2Ks) (Robinson and Cobb 1997). Whereas the MAP2K4 and MAP2K7 are the upstream kinases for JNK activation, the MAP2K3 and MAP2K6 are specific for p38, and the MAP2K1 and MAP2K2 are specific for ERK activation.

Once activated, the MAPKs can translocate to the nucleus, where they phosphorylate transcription factors causing gene expression changes. The activated MAPKs exhibit distinct specificity in nuclear transcription factor phosphorylation. For instance, JNKs are the upstream kinases for c-JUN, whereas both JNK and p38 can phosphorylate ATF2; the ERKs, on the other hand, specifically catalyze the phosphorylation of ELK1. Through the distinct connections to upstream regulators and downstream effectors, each MAPK exhibits unique roles in converting transient biochemical signals to permanent changes of gene expression in the control of cell functions.

The existing literature describes divergent effects of Cr(VI) on MAPK signaling, as activation patterns vary depending on different experimental systems, treatment concentrations, time of exposure, and target cell type used for the studies (Chuang and Yang 2001; O’Hara et al. 2003; Samet et al. 1998; Tessier and Pascal 2006). Several studies show that high concentrations of Cr(VI) can activate all three MAP kinases and that the activation may be mediated via reactive oxygen species (ROS)-dependent mechanisms. Conversely, lower concentrations of Cr(VI) may activate selective MAPK subgroups in a highly cell-type-specific fashion, whereas activation might be ROS independent. Induction of MAP kinase pathways was shown to contribute to specific toxic responses to Cr(VI) (Wakeman et al. 2005; Wang and Shi 2001), although in other cases could not be linked to a defined end point of Cr(VI) toxicity (Ceryak et al. 2004; Chuang and Yang 2001). Collectively, these observations suggest that MAPK activation is due to the combinatorial effects of Cr(VI) bioprocessing and dynamic interactions of Cr species with other cellular components within the local environment. As a consequence, the contribution of each MAPK pathway to chromium toxicity will be better evaluated individually in the specific cellular context and exposure condition. In this paper, we characterized the effects of high and low concentrations of Cr(VI) on MAPK activation profiles in wild-type (WT) and *Map2k4*^(−/−)^ and *Map2k7*^(−/−)^ embryonic stem (ES) cells. Using these cells, we show that Cr(VI) activates all three MAPKs, but each of them leads to specific transcription factor modifications and makes unique contributions to Cr(VI) cytotoxicity and differentiation inhibition.

## Materials and Methods

### Reagents and antibodies

Dulbecco’s minimum essential medium (DMEM), l-glutamine, MEM nonessential amino acids, and penicillin-streptomycin were purchased from Mediatech (Manassas, VA); Eagle’s MEM was from Lonza Inc **(**Basel, Switzerland**)**; ESGRO (LIF) was from Millipore **(**Temecula, CA**)**; calcium chloride was from Cascade Biologics **(**Invitrogen; Carlsbad, CA**)**; and fetal bovine serum (FBS) knockout serum replacement, β-mercaptoethanol, and CM-H_2_ 5′,6′-chloromethyl-2′-7′-dichlorofluorescin diacetate (DCFDA) were purchased from Invitrogen. The chemical inhibitors for JNK (SP600125), p38 (SB202190), ERK (PD98059), and protein synthesis [cycloheximide (CHX)] were from Calbiochem **(**Gibbstown, NJ**)**; *N*-acetyl cysteine (NAC) and Cr(VI) were from Sigma **(**St. Louis, MO**)**. Adenoviruses for GFP and MAP2K7 were as described previously (Deng et al. 2006). Antibodies for phospho-JNK (Thr-183, Tyr-185) and total JNK, phospho-p38 (Thr-180, Tyr-182) and total p38, phospho-ERK (Thr-202, Thr-204) and total ERK, as well as antibodies for phospho-c-Jun (Ser-63), phospho-ATF2 (Thr-69,71), poly-(ADP-ribose) polymerase (PARP), MAP2K4, and MAP2K7 were purchased from Cell Signaling Technology **(**Danvers, MA**)**; antibodies for total c-Jun, phospho-ELK1 (Ser-383), and c-FOS were from Santa Cruz Biotechnology **(**Santa Cruz, CA**)**; anti bodies for β-actin and active caspase-3 were from Pharmingen **(**San Diego, CA**)**, and anti-GFP was from Roche **(**Palo Alto, CA**)**.

### Cell culture and adenoviral infection

We precoated culture dishes used for mouse ES cells with 0.1% gelatin for 20 min. The ES cells of WT, *Map2k4*^(−/−)^, and *Map2k7*^(−/−)^ were maintained in ES complete medium, containing DMEM, 15% FBS knockout serum replacement, 2 mM l-glutamine, 50 U/mL penicillin, 50 μg/mL streptomycin, 1% nonessential amino acids, 0.1mM β-mercaptoethanol, and 10^3^ units/μL LIF. We carried out infection of the ES cells with adenovirus as described previously (Deng et al. 2006). At 48 hr after infection, we subjected the cells to subsequent treatments with Cr(VI) or harvested them for Western blot analyses.

### Cytotoxicity assay

Acute cytotoxicity assays were performed on ES cells seeded at 1.5 × 10^4^ cells/well on 96-well plates pre-coated with 0.1% gelatin. We treated the cells with various concentrations of Cr(VI), and measured the cytotoxicity using Cell Titer 96 AQ_ueous_ One Solution [MTS; 3-(4,5-dimethylthiazol-2-yl)-5-(3-carboxymethoxyphenyl)-2-(4-sulfophenyl)-2H-tetrazolium] following the manufacture’s recommended procedures (Promega, Madison, WI).

Chronic toxicity assay was performed as described (Franken et al. 2006). Briefly, we plated 5,000 ES cells onto gelatin pretreated 6-mm plates in ES complete medium in the absence and presence of various doses of Cr(VI). Cells were allowed to grow for 7 days, and the ES complete medium was replenished every 3 days. We washed the colonies with phosphate-buffered saline, fixed, and stained them with 0.3% crystal violet mixed in 25% ethanol for 2 min and counted them under the microscope. We used three plates for each treatment condition.

### Cell lysate preparation and immunoblotting

ES cells were challenged with Cr(VI) at various conditions described above. Cell lysates were prepared and subjected to SDS-PAGE and transferred to nitrocellulose membranes for Western blotting analyses as described elsewhere (Peng et al. 2007).

### Cellular ROS and glutathione measurement

We measured the ROS levels in the cells by CM-H_2_ DCFDA labeling, and identified the fluorescence-positive cells by FACS analyses as described previously (Peng et al. 2007). Glutathione and glutathione disulfide (GSSG) levels were determined as detailed previously (Senft et al. 2002), with samples prepared in triplicate for statistical analysis.

### Differentiation of ES cells into contracting myocardial cells

We conducted cardiac differentiation of ES cells as described previously (Gribaldo et al. 2002). On day 0, we aggregated ES cells (5 × 10^4^ cells/mL) by culturing in 20-μL hanging drops in cell culture medium without LIF. On day 3, the cell aggregates formed embryoid bodies (EBs), and the EBs were transferred to 24-well plates at 1 EB/well. On day 14, we counted the number of EBs with contracting myocardial areas under light microscopy. In some experiments, Cr(VI) was included in the growth medium for the entire time of differentiation. We replenished the culture medium with or without chemicals every 3 days.

### Real-time polymerase chain reaction

We isolated total cellular RNA from WT and *Map2k7*^(−/−)^ ES cells and EBs, which were allowed to differentiate for 14 days in the absence and presence of 0.1 μM Cr(VI). We performed cDNA preparation and real-time PCR as described previously (Peng et al. 2007). Primers for mouse stem-cell-specific genes, *Notch-1*: 5′-AAT CTT GTC CAG ACA GGT GCC AGA and 5′-TCT GCT TAT GCC TCA AGG GAA CCA, *Sox-2*: 5′-AAA GGA GAG AAG TTT GGA GCC CGA and 5′-GGG CGA AGT GCA ATT GGG ATG AAA, *Oct-4:* 5′-GGC GTT CTC TTT GGA AAG GTG TTC and 5′-CTC GAA CCA CAT CCT TCT CT and *Nanog*: 5′-AGG GTC TGC TAC TGA GAT GCT CTG and 5′-CAA CCA CTG GTT TTT CTG CCA CCG; for cardiomyocyte-specific genes, *Mhca*: 5′-CTG CTG GAG AGG TTA TTC CTC G and 5′-GGA AGA GTG AGC GGC GCA TCA AGG and *Mhcb*: 5′-TGC AAA GGC TCC AGG TCT GAG GGC and 5′-GCC AAC ACC AAC CTG TCC AAG TTC; and for housekeeping genes, *Actinb*: 5′-TCT TGG GTA TGG AAT CCT GTG GCA and 5′-TCT CCT TCT GCA TCC TGT CAG CAA and *Gapdh*: 5′-TCA ACA GCA ACT CCC ACT CTT CCA and 5′-ACC CTG TTG CTG TAG CCG TAT TCA were used for RT-PCR.

### Statistical analyses

We conducted statistical comparisons using Student two-tailed paired *t -* test and analysis of variance. We considered values of *p*< 0.05 statistically significant.

## Results

### High-concentration Cr(VI) activates MAPKs and downstream transcription factors in mouse ES cells

**Figure 1:**
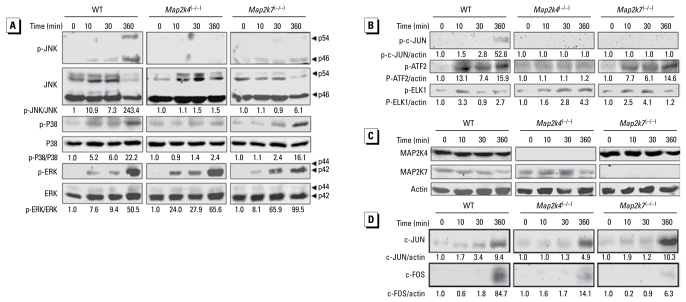
High-concentration Cr(VI) induces MAPK activation in WT, *Map2k4*^(−/−)^*,* and *Map2k7*^(−/−)^ mouse ES cells. WT, *Map2k4*^(−/−)^*,* and *Map2k7*^(−/−)^ cells were treated with 50 μM Cr(VI) for various times as indicated. Cell lysates were subjected to Western blot analysis for (*A*) phospho-JNK and total JNK, p38, and ERK; (*B*) phospho-c-JUN, ELK1, and ATF2; (*C*) total MAP2K4, MAP2K7, and actin; and (*D*) total c-JUN and c-FOS. Arrowheads point to specific MAPK isoforms. The ratio of phosphoprotein versus total protein or actin was calculated in each sample and the fold induction was calculated in comparison with the untreated control of the same cell type. Images shown in this figure are representative of at least two independent results.

We examined MAPK activation in mouse ES cells treated with Cr(VI) at 50 μM for various lengths of time. Cr(VI) caused early and delayed ERK activation, as indicated by phospho-ERK2 (p42) induction, and delayed JNK and p38 activation, as indicated by the appearance of phospho-JNK (p54 and p46) and phospho-p38 (Figure 1A). Correspondingly, Cr(VI) induced the MAPK downstream events, including phosphorylation of c-JUN, ATF2, and ELK1 in WT ES cells (Figure 1B). In addition, there was an apparent elevation of c-JUN and c-FOS proteins at the delayed phase of exposure (Figure 1D).

In recent years, genetic inactivation of MAP2Ks has been shown to specifically block MAP kinase activation, and the MAP2K-deficient cells are used to sort out the roles of the MAPKs in variety of cell functions (Wada et al. 2008). We examined MAPK activation by Cr(VI) in the MAP2K4-deficient and MAP2K7-deficient ES cells (Figure 1C). Cr(VI) exposure did not alter MAP2K4/MAP2K7 expression. Compared with the induction in the WT cells, p-JNK and p-p38 induction by Cr(VI) in the *Map2k4*^(−/−)^ cells was almost completely abolished [Figure 1A; see Supplemental Material, Figure 1 (available online at http://www.ehponline.org/members/2009/0800157/suppl.pdf)]. Correspondingly, there were no detectable p-c-JUN and activating transcription factor-2 (p-ATF-2) in the *Map2k4*^(−/−)^ cells [Figure 1B; see Supplemental Material, Figure 1 (http://www.ehponline.org/members/2009/0800157/suppl.pdf)]. The phosphorylation of JNK and c-JUN was markedly suppressed in the *Map2k7*^(−/−)^ cells, whereas the phosphorylation of p38 and ATF-2 was unaffected. The phosphorylation of ERK and ELK1 were not overtly affected by MAP2K4-ablation and MAP2K7-ablation, indicating that these MAP2Ks were not essential for ERK pathway activation by Cr(VI) (Figures 1A and 1B). Based on these observations, we conclude that the Cr(VI) signals that activate JNK are mediated by both MAP2K4 and MAP2K7 and those that activate p38 by MAP2K4; however, neither MAP2K4 nor MAP2K7 are required for ERK activation and the induction of c-JUN and c-FOS expression (Figure 1D).

### MAP2K7 and JNK reduce, but p38 promotes, cytotoxicity induced by high concentration of Cr(VI)

**Figure 2:**
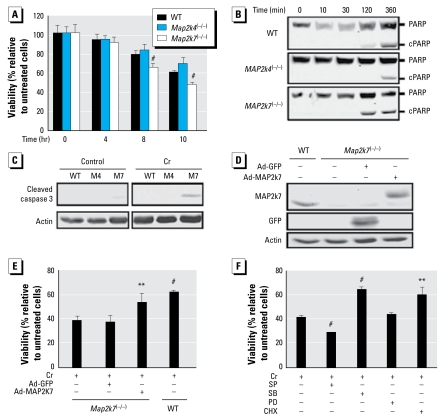
MAP2K7 and JNK protect cells from cytotoxicity from high-concentration Cr(VI). WT, *Map2k4*^(−/−)^*,* and *Map2k7*^(−/−)^ ES cells were treated with 50 μM Cr(VI) for different times as indicated. (*A*) MTS assay was performed to evaluate cell survival. Cell lysates were subjected to Western blotting to monitor the levels of apoptotic markers; (*B*) PARP cleavage and (*C*) active caspase-3. *Map2k7*^(−/−)^cells were infected with adenoviruses for either GFP or MAP2K7 at 100 pfu/cell. At 48 hr of infection, (*D*) the expression of MAP2K7, GFP, and actin in WT, *Map2k7*^(−/−)^*,* and *Map2k7*^(−/−)^ adenoviral-infected cells were examined by Western blotting. (*E*) WT, *Map2k7*^(−/−)^*,* and *Map2k7*^(−/−)^ adenoviral-infected cells were treated with 50 μM Cr(VI) for 10 hr. Cell survival was determined by MTS assays, and each value was presented as mean ± SD from four replicates. (*F*) WT ES cells were pretreated with chemical inhibitor for JNK (SP60015, 5 μM), p38 (SB202190, 5 μM), ERK (PD98059, 5 μM), and protein synthesis (CHX, 1 μg/mL) for 1 hr. The cells were incubated with Cr(VI) at 50 μM for 8 hr. Cell survival rates were analyzed by MTS assay, and each value was presented as mean ± SD from four replicates. **p < 0.01. ^#^*p <* 0.001.

Cr(VI) cytotoxicity in WT ES cells depended on concentration and time of exposure and was significantly increased by treatment with 50 μM Cr(VI) for ≥ 6 hr [see Supplemental Material, Figures 1 and 2 (http://www.ehponline.org/members/2009/0800157/suppl.pdf)]. In comparison with WT cells, *Map2k4*^(−/−)^ cells had a slight but insignificant reduction of cytotoxicity. Conversely, *Map2k7*^(−/−)^ cells had a marked increase in cytotoxicity (Figure 2A). The increased cytotoxicity of *Map2k7*^(−/−)^ cells correlated with higher levels of PARP cleavage and active forms of caspase-3, both markers of apoptosis activation (Figures 2B and 2C).

To confirm that MAP2K7 conferred resistance to Cr(VI) cytotoxicity, we over-expressed MAP2K7 in *Map2k7*^(−/−)^ cells using an adenovirus-mediated gene delivery system. At 48 hr of infection, the levels of MAP2K7 in Ad-MAP2K7-infected, but not Ad-GFP infected, *Map2k7*^(−/−)^ cells were similar to or even higher than those in the WT cells (Figure 2D). When exposed to Cr(VI), the Ad-MAP2K7-infected *Map2k7*^(−/−)^ cells reached a survival rate similar to that of the WT cells, much higher than the parental uninfected or Ad-GFP-infected cells (Figure 2E).

To test whether MAPK activation was responsible for Cr(VI) cytotoxicity, we treated the WT ES cells with specific MAPK inhibitors prior to Cr(VI) exposure. Our previous work had established that at 5 μM concentration short-term treatment, SP600125 was specific for JNK inhibition, and similarly, SB202190 was specific for p38 and PD98059 for ERK (Deng et al. 2006). Whereas inhibition of JNK sensitized the cells to Cr(VI) cytotoxicity, inhibition of p38 reduced it, and inhibition of ERK had no effect (Figure 2F). Furthermore, pre-treatment of cells with CHX to inhibit protein synthesis significantly reduced, but did not completely prevent, Cr(VI) cytotoxicity in WT ES cells (Figure 2F), suggesting that gene transcription and de novo protein synthesis contribute partially to Cr(VI) cytotoxicity. Considering that *Map2k7*^(−/−)^ cells are defective in JNK activation and are more sensitive to Cr(VI) cytotoxicity, a condition similar to that of WT cells treated with the JNK inhibitors, we suggest that JNK activation is required for reducing Cr(VI) cytotoxicity. On the other hand, cells treated with the p38 inhibitor and *Map2k4*^(−/−)^ cells, defective in p38 activity, are both somewhat Cr-resistant, suggesting that p38 activation may augment Cr(VI) cytotoxicity.

### N-acetyl cysteine partially blocks high-concentration Cr(VI)-induced MAPK activation and cytotoxicity

**Figure 3:**
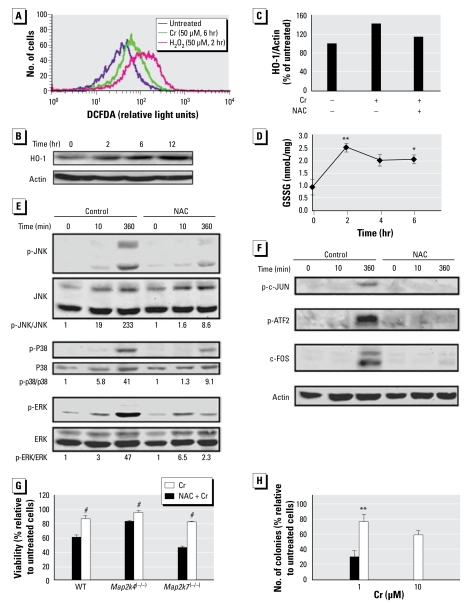
NAC suppresses MAPK activation and cytotoxicity induced by high-concentration Cr(VI). (*A*) WT ES cells were treated with 50 μM Cr(VI) for 6 hr or 50 μM H_2_O_2_ for 2 hr. The cells were labeled with 10 μM CM-H_2_DCFDA for 30 min and were subjected to flow cytometric analysis. DCF-positive cells were identified by CellQuest analysis. Cells were either pretreated with 5 mM NAC for 4 hr or left untreated prior to exposure to 50 μM Cr(VI) for various times. (*B*) Western blotting was used to measure the expression of HO-1 and actin in WT ES cells treated with Cr(VI) 50 μM for various times. (*C*) The relative levels of HO-1 protein expression were quantified by gel imaging in ES cells with or without NAC and Cr(VI) treatment. (*D*) Cells exposed to Cr(VI) at 50 μM for various times were analyzed for the cellular level of GSSG. Western blot was done to measure (*E*) phosphorylated and total JNK, p38, and ERK and (*F*) p-JUN, p-ATF2, c-FOS, and actin. The results were quantified by chemiluminescence imaging and the fold induction of p-JNK over JNK, p-38 over P38, p-ERK over ERK, and p-c-Jun, p-ATF2, and c-Fos over actin was calculated. (*G*) WT, *Map2k4*^(−/−)^*,* and *Map2k7*^(−/−)^ ES cells were either pretreated with 5 mM NAC for 4 hr or left untreated prior to exposure to 50 μM Cr(VI) for 8 hr. Cell survival was measured by the MTS assay. (*H*) WT cells were exposed to 1 μM or 10 μM Cr(VI) in the presence or absence of 5 mM NAC. Colonies were counted on day 8 of treatment. The values in B, D, and E are shown as mean ± SD from at least three experiments. The ratio of phosphoprotein versus total protein or actin was calculated in each sample, and the fold induction was calculated in comparison with the untreated control of the same cell type. *p < 0.05. **p < 0.01. ^#^*p <* 0.001.

It has been suggested that Cr(VI) can activate MAPKs via ROS-dependent and ROS-independent mechanisms (Kim and Yurkow 1996; O’Hara et al. 2003). We labeled the WT ES cells, either untreated or treated with Cr(VI) and H_2_O_2_, with ROS-activated fluorescent dye CM-H_2_DCFDA and measured intracellular ROS levels by flow cytometry. Compared with the untreated cells, ROS accumulation was higher in Cr(VI)-treated cells, but the levels induced by Cr(VI) were lower than those induced by H_2_O_2_ (Figure 3A). *Map2k4*^(−/−)^ and *Map2k7*^(−/−)^ ES cells displayed similar patterns of ROS induction by Cr(VI) [see Supplemental Material, Figure 4 (http://www.ehponline.org/members/2009/0800157/suppl.pdf)]. To further evaluate the ROS induction by Cr(VI), we measured the expression of heme oxygenase-1 (HO-1), a known marker of cellular oxidative stress. Cr(VI), in an exposure time-dependent manner, caused an apparent HO-1 induction, which was partially blocked by pretreatment with NAC, an antioxidant that elevates glutathione (GSH) and prevents ROS accumulation (Figures 3B and 3C). Cellular redox homeostasis is largely dependent on GSH, which when oxidized by ROS, forms GSSG. We found higher GSSG in ES cells treated by Cr(VI) than in untreated cells, further supporting the idea that Cr(VI) induced oxidative stress and caused GSH depletion (Figure 3D)

To test whether NAC can prevent MAPK activation and cytotoxicity in our system, we treated WT cells with NAC prior to Cr(VI) exposure. In NAC pretreated cells, there was a significant decrease in the induction of p-JNK, p-p38, and p-ERK by Cr(VI) at the late exposure times (6 hr), but induction of p-ERK at early times (10 min) did not seem to be affected (Figure 3E). Induction of the nuclear factors p-JUN, p-ATF2, and c-FOS was also significantly reduced by NAC pretreatment (Figure 3F). Moreover, NAC significantly prevented Cr(VI) cytotoxicity as measured by both the acute and chronic toxicity (Figures 3G and 3H). Protection by NAC was much more effective than by MAPK inhibitors or by MAP2K gene ablation, indicating that NAC offers a broader protection against Cr(VI) cytotoxicity and that one of the downstream events of NAC may be to reduce the delayed MAPK activation.

### The role of MAPKs in low-concentration Cr(VI) effects

**Figure 4:**
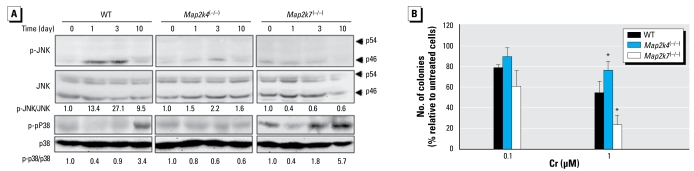
MAPK activation and cytotoxicity in response to low-concentration Cr(VI). (*A*) WT, *Map2k4*^(−/−)^*,* and *Map2k7*^(−/−)^ ES cells were treated with 0.1 μM Cr(VI) as indicated. Cell lysates were used for Western blot analyses for the phospho-JNK and total JNK and p38. Arrowheads point at different JNK isoforms. The ratio of phosphoprotein versus total protein was calculated in each sample, and the fold induction was calculated in comparison with the untreated control of the same cell type. (*B* )Cells were treated with 0.1 μM or 1 μM Cr(VI) for 7 days, and colonies were counted on day 8 of treatment. Each value represents the mean ± SD from six replicates. *p < 0.05.

Having established the MAPK activation patterns in response to high Cr(VI) concentrations, we asked whether the MAPKs, particularly JNK and p38, were similarly affected by MAP2K4/MAP2K7 ablation in response to low concentrations of Cr(VI), more comparable with potential environmental or occupational human exposures. We treated the ES cells with 0.1 μM Cr(VI) for various times ranging from 1 to 10 days and examined the phospho-JNK and total-JNK and p38 (Figure 4A). Low-concentration Cr(VI) caused a clear induction of p-JNK in WT ES cells. In contrast to high-concentration Cr(VI), which strongly induced phosphorylation of both p46 and p54 JNK isoforms (Figure 1A), low-concentration Cr(VI) only weakly activated the p46 JNK isoform. Similarly, low-concentration Cr(VI) induced weaker and delayed p38 phosphorylation. JNK activation by low-concentration Cr(VI) was completely abolished in *Map2k4*^(−/−)^ and *Map2k7*^(−/−)^ cells, whereas p38 activation was unaffected in *Map2k7*^(−/−)^ but blocked in *Map2k4*^(−/−)^ cells, similar to the activation patterns in response to high concentration (Figure 1A).

We next examined the contributions of MAPKs to chronic toxicity of Cr(VI) using colony formation assays (Figure 4B). In comparison with the WT ES cells, *Map2k4*^(−/−)^ cells showed significantly higher chronic survival rate in response to 1 μM Cr(VI) treatment for 7 days, whereas *Map2k7*^(−/−)^ cells had markedly decrease survival rate. Based on these observations, we conclude that, like high-concentration Cr(VI), low concentration activates JNK through MAP2K4/7 and p38 through MAP2K4 in ES cells and that MAP2K4 and MAP2K7 have similar effect on the cytotoxicity of low and high concentrations of Cr(VI).

### Suppression of ES cell differentiation by chromium requires MAP2K7

**Figure 5:**
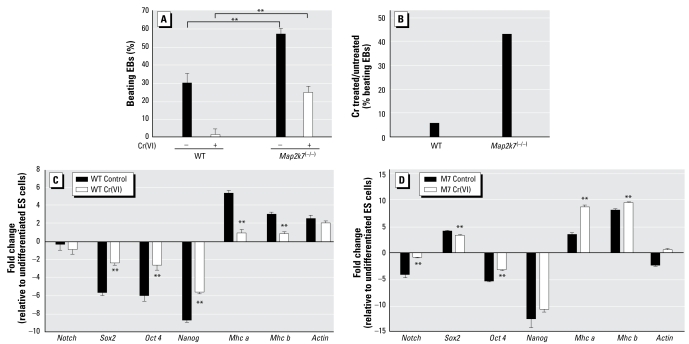
MAP2K7-deficient cells are resistant to Cr(VI) developmental toxicity. WT and *Map2k7*^(−/−)^ EBs were grown in the presence or absence of 0.2 μM Cr(VI) for 8–13 days. The percentage of EBs that developed rhythmic beating cardiomyocytes (*A*) and the percentage inhibition of beating cardiomyocytes formation by Cr(VI) (*B*) were calculated. The values are shown as mean ± SD from at least three experiments. In WT (*C*) and *Map2k7*^(−/−)^ (*D*) EBs, the relative levels of stem-cell-specific and cardiomyocyte-specific genes over *Gapdh* were calculated. The values are shown as mean ± SD from at least three experiments. **p < 0.01.

Developing rhythmically contractile cardiomyocytes from EBs were used to evaluate the developmental effects of MAPKs and Cr(VI). Although 30% of WT EBs and 60% of MAP2K7-deficient EBs developed rhythmic contractile cardiomyocytes, none of the MAP2K4-deficient EBs formed them (Figure 5A and data not shown). Because p38 α-deficient ES cells have failed cardiomyocyte differentiation, it is possible that the MAP2K4-deficient cells lack p38 activation and therefore have a defective cardiac differentiation (Aouadi et al. 2006). Cr(VI) at 1 μM completely abolished WT EB to form contractile cardiomyocytes, but at 0.01 μM had no effect at all (data not shown). At 0.1 μM, Cr(VI) caused a 95% suppression of cardiac differentiation in WT EBs, but less than 50% suppression in MAP2K7-deficient EBs. Based on these evaluations, we conclude that MAP2K7 inactivation might offer protection against Cr(VI) toxicity in the inhibition of ES cell differentiation (Figures 5A and 5B).

To study EB differentiation at the molecular level, we examined the expression of stem-cell-specific and cardiomyocyte-specific markers by RT-PCR. Compared with undifferentiated ES cells, the differentiated WT and *Map2k7*^(−/−)^ EBs had decreased expression of stem-cell-specific genes, such as *Notch*, *Sox-2*, *Nanog,* and *Oct-4*, but elevated expression of cardiomyocyte genes, such as *MhcA* and *MhcB*, indicative of cardiomyocyte differentiation (Figures 5C and 5D). Such changes in gene expression were clearly suppressed by Cr(VI) in WT but not in *Map2k7*^(−/−)^ EBs. Thus, this gene expression signature also suggests that MAP2K7 inactivation prevents the inhibitory effects of Cr(VI) on ES cell differentiation.

## Discussion

**Figure 6:**
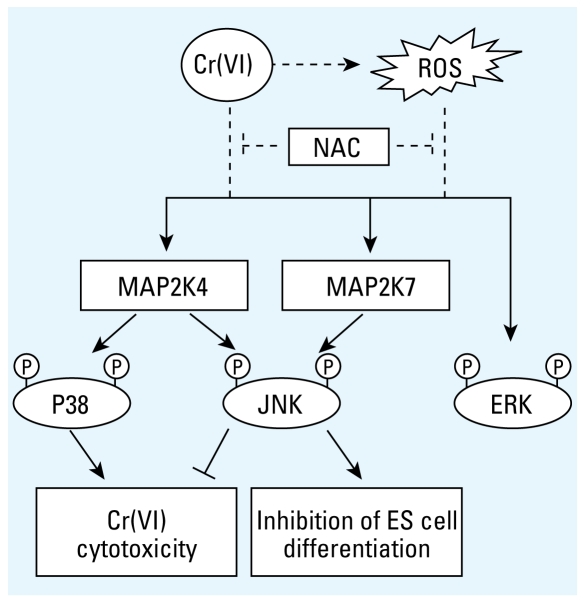
Distinct contributions of JNK and p38 to Cr(VI) toxicity. Cr(VI) induced the activation of the MAPKs via multiple mechanisms that can be ROS dependent and independent. The activation of JNK and p38, but not ERK, is mediated through MAP2K4 and MAP2K7. Specifically, MAP2K4 and MAP2K7 both are required for optimal JNK activation, but only MAP2K4 is essential for p38 activation. Using cells deficient in MAP2K4 and MAP2K7, we were able to delineate the distinct roles JNK and p38 play in the cytotoxicity and developmental toxicity of chromium.

Cr(VI) exposure is associated with broad classes of adverse health effects in humans and in animal models, including respiratory cancer, impaired organ function, immune system defects, birth defects, and decreased fertility (Junaid et al. 1996; Kanojia et al. 1998; Salnikow and Zhitkovich 2008), but the molecular mechanisms underlying disease development are not well understood. In the present studies, we show that one consequence of Cr(VI) exposure of mouse ES cells is the early and delayed activation of MAPKs. There is a good correlation between the MAPKs and their corresponding downstream transcription factor modifications. These results, together with the findings that Cr(VI) cytotoxicity requires *de novo* protein synthesis, indicate that the MAPKs may activate transcriptional programs for gene expression, which in turn influences cell fate determination in response to Cr(VI) (Figure 6).

Similar MAPK induction by Cr(VI) has been observed in various cells types (Ceryak et al. 2004; Chuang and Yang 2001; O’Hara et al. 2003; Samet et al. 1998; Tessier and Pascal 2006; Wakeman et al. 2005), but the specific contributions of the different MAPKs to Cr(VI) toxicity are poorly defined. Studies based solely on pharmaceutical MAPK inhibitors used at high concentrations that have been shown to cause nonspecific effects (Tan et al. 2002) often generate diametrically opposite results (Chuang and Yang 2001; Wakeman et al. 2005). We used instead the combined approach of MAP2K-deficient cells and low-dose MAPK inhibitors for short-term treatment to reveal the distinct contributions each MAPK makes to acute Cr(VI) cytotoxicity. We show that JNK alleviates, p38 promotes, and ERK has no obvious effect on cytotoxicity of Cr(VI) at high and low concentrations (Figure 6). Antagonistic roles of JNK and p38 have been described previously, in that JNK positively regulates proliferation and oncogenic transformation but negatively regulates cellular senescence, whereas p38 suppresses proliferation and transformation but potentiates cellular senescence (Wada et al. 2008). Our studies for the first time report opposing effects of JNK and p38 exist for Cr(VI) cytotoxicity.

In addition to its acute cytotoxicity, Cr(VI) is a known developmental toxicant, causing postimplantation losses, resorptions, reduced fetal weight, and malformations including reduced ossification (Junaid et al. 1996; Kanojia et al. 1998). We have found that Cr(VI) is a strong developmental toxicant that, at concentrations as low as 0.1 μM, causes 95% inhibition of cardiac differentiation of EBs, corresponding to the repression of cardiomyocyte-specific gene induction. This observation is in general agreement with the findings by Stummann et. al. showing that 1.8 μM Cr(VI) causes 50% inhibition of the cardiac differentiation of D3 ES cells (Stummann et al. 2007). However, the ES cell lines used in our studies appear to be even more sensitive to Cr(VI), perhaps because they originated from the Sv129 mouse strain.

Although the chemical inhibitors for short-term treatment can effectively block MAPK activity and cytotoxicity of chromium, the JNK inhibitor SP600125 completely abrogated the EB formation when used for a longer treatment period (data not shown). These observations suggest that long-term application of these chemicals may exhibit nonspecific toxicity irrelevant to JNK inhibition, because *in vivo* JNK1/JNK2 double ablation does not cause global differentiation defects (Kuan et al. 1999).

Because JNK is the only known downstream target of MAP2K7, the *Map2k7*^(−/−)^ cells have been conveniently used to evaluate physiologic consequences of JNK loss of function (Wada et al. 2008). Indeed, we show that JNK is the sole MAPK whose activation by Cr(VI) is effectively abrogated in *Map2k7*^(−/−)^ ES cells. Intriguingly, Cr(VI) causes only a slight suppression of contractile cardiomyocyte formation in the *Map2k7*^(−/−)^ EBs and does not cause reduction of cardiomyocyte-specific gene expression in these cells. Because Cr(VI) fails to activate JNK in *Map2k7*^(−/−)^ cells, it is possible that blocking JNK reduces Cr(VI) toxicity on the inhibition of cardiomyocyte differentiation. The seemingly contrasting role that the JNK pathway appears to have by inhibiting cardiomyocyte differentiation on the one hand and preventing acute and chronic cytotoxicity on the other is not entirely surprising, because the JNK pathway is known to regulate diverse transcriptional programs, participating in a broad spectrum of cell responses.

The MAP2Ks are immediately upstream kinases responsible for MAPK phosphorylation and activation. It has been shown that MAP2K4 and MAP2K7 distinctly phosphorylate JNK at tyrosine and threonine, respectively (Lawler et al. 1998). We find that Cr(VI) reduces JNK phosphorylation and completely abolished c-JUN phosphorylation in *Map2k4*^(−/−)^ and *Map2k7*^(−/−)^ cells. This observation seems to support the notion that MAP2K4 and MAP2K7 phosphorylate distinct sites at the Thr-X-Tyr motif of JNK and that phosphorylation of both tyrosine and threonine residues is required for optimal JNK activity in c-JUN phosphorylation. Besides JNK activation, MAP2K4 is also known to activate p38. In ES cells, MAP2K4 appears to be essential for p38 activation by Cr(VI), even though these cells have other p38 MAP2Ks, such as MAP2K3 and MAP2K6 (data not shown). It remains to be determined, however, whether MAP2K4 activates p38 directly or indirectly through the activation of MAP2K3/6 in the transmission of Cr(VI) signals. Under *in vivo* physiologic conditions, the MAP2K4 and MAP2K7 have nonredundant roles in fetal liver development (Wang et al. 2007). Our *in vitro* studies show that *Map2k4*^(−/−)^ ES cells fail to develop beating cardiomyocytes. On the other hand, *Map2k7*^(−/−)^ ES cells form beating cardiomyocytes more efficiently than the WT cells, suggesting that MAP2K7 suppresses cardiac differentiation.

Cr(VI) undergoes intracellular reduction, resulting in the generation of toxic Cr intermediates and thermodynamically stable Cr(III), which are ultimately responsible for DNA damage (Salnikow and Zhitkovich 2008), and of ROS, which contribute to cytotoxicity. Cr(VI) reduction is carried out by cellular antioxidant systems such as glutathione and ascorbate. Ascorbate has been shown to increase Cr(VI) reductive activation, enhance toxicity, and potentiate mutagenicity, suggesting that ascorbate, commonly used as a redox scavenger to prevent toxicity of other environmental agents, is not suitable for counteracting Cr(VI) toxicity (Salnikow and Zhitkovich 2008). We find that incubation of cells in ascorbate at 1 mM for a shorter time (< 12 hr) offers no protection against Cr(VI) cytotoxicity, but for a long time (> 20 hr) is by itself toxic to ES cells even in the absence of Cr(VI) (data not shown). This observation precludes the possibility of further evaluating the role of ascorbate in Cr(VI) cytotoxicity in our experimental systems. On the other hand, NAC exhibits a remarkable protection against Cr(VI) cytotoxicity. Furthermore, inclusion of 5 mM NAC in the growth medium does not completely abrogate, but significantly reduces, the delayed MAPK activation by Cr(VI). There are multiple possibilities for NAC to exert protection, including potentiation of Cr(VI) extracellular bioreduction, elevation of intracellular GSH to promote the removal of ROS, and promotion of electron transfer for direct Cr(VI) intracellular reduction (Guttmann et al. 2008; Salnikow and Zhitkovich 2008), all of which could be the reason for NAC to decrease Cr(VI)-induced MAPK activity. It is worth noting that EB and contractile cardiomyocyte formation are completely abrogated in the presence of 0.5 mM NAC in the medium (data not shown), indicating that excess cysteine can also disrupt normal ES cell differentiation programs.

Our understanding of the molecular events mediating cellular responses to Cr(VI) is rapidly expanding. In addition to causing DNA damage and modifying chromatin, Cr(VI) can induce MAPKs to affect the well-being of the organism. Moreover, we find that each MAPK member makes a distinct contribution to Cr(VI) cytotoxicity and inhibition of differentiation (Figure 6). The experimental systems described in our studies may provide useful tools to evaluate the contributions of the MAPK members to other Cr(VI) toxic end points, such as cell cycle progression, differentiation, development, and movement.
